# Change in the Acceptance of Telemedicine Use Among Older Patients with Knee Osteoarthritis During the Coronavirus Disease 2019 Pandemic

**DOI:** 10.1089/tmr.2021.0052

**Published:** 2022-02-14

**Authors:** Tsuneari Takahashi, Ryusuke Ae, Koki Kosami, Kensuke Minami, Meiwa Shibata, Tatsuya Kubo, Katsushi Takeshita

**Affiliations:** ^1^ Department of Orthopaedic Surgery, School of Medicine, Jichi Medical University, Shimotsuke, Japan.; ^2^ Division of Public Health, Center for Community Medicine, Jichi Medical University, Shimotsuke, Japan.; ^3^ Department of Infection Control, School of Medicine, Jichi Medical University, Shimotsuke, Japan.; ^4^ Division of Infectious Diseases, Tokyo Metropolitan Children's Medical Center, Tokyo, Japan.; ^5^ Gunma Sports Medicine Research Center, Zenshukai Hospital, Maebashi, Japan.

**Keywords:** acceptance, coronavirus disease 2019, knee osteoarthritis, orthopedic practice, outpatient, telemedicine

## Abstract

**Background:** Hospital-related coronavirus disease 2019 (COVID-19) infection is of utmost concern among patients and health care workers. Expanding the use of telemedicine may be required in daily outpatient practice; however, the acceptance of telemedicine use is still low, especially among older patients. In an orthopedic practice, no studies have investigated potential factors that can contribute to changes in the acceptance of using telemedicine. Focusing on older outpatients with knee osteoarthritis (KOA), we hypothesized that a drastic surge in the number of patients with COVID-19 could trigger changes in attitudes regarding the acceptance of telemedicine use.

**Methods:** A baseline survey was conducted after the first wave of the COVID-19 pandemic in Japan to obtain information on the willingness to use telemedicine among patients aged ≥70 years who regularly consulted an orthopedic surgeon for KOA. A follow-up survey was subsequently conducted during the third wave of the pandemic period to assess changes in the acceptance of telemedicine use in response to the rapidly increasing number of patients with COVID-19. We compared the difference in acceptance of telemedicine use and knee pain status between the baseline and follow-up surveys.

**Results:** In the baseline survey, 11 of 43 patients (25.6%) responded that they would be willing to use telemedicine. In the follow-up survey, patients' acceptance of telemedicine did not change, with the exact same number and percentage of patients who were willing to use telemedicine as in the baseline survey, despite that ∼20% of patients reported improvement in their knee pain status.

**Discussion:** Our findings indicate that older outpatients with KOA did not change their willingness to accept use of telemedicine, even with a drastically increased risk of hospital-related transmission of a potentially fatal infectious disease when visiting a hospital. The acceptance of telemedicine use among older patients might not be less sensitive to external environmental factors but instead might be more sensitive to patients' personal factors, such as anxiety for information technology and resistance to changes in their lifestyle.

## Introduction

The outbreak of severe acute respiratory syndrome coronavirus 2 (SARS-CoV-2) has spread worldwide.^1^ SARS-CoV-2 infection in patients who develop coronavirus disease 2019 (COVID-19) may be more infectious than those with other acute respiratory syndromes, even though some infected individuals are asymptomatic.^1^ Hospital-related transmission of SARS-CoV-2^2^ is of utmost concern among patients and health care workers.^3,4^ Because the fatality rate of COVID-19 is estimated to be higher among patients aged ≥70 years,^5^ asymptomatic transmission of SARS-CoV-2 in health care institutions may adversely affect the health of older patients who regularly consult a physician in a medical institution.

Although the use of telemedicine has increased over the past 2–3 years,^6^ only a few patients have adopted it for orthopedic consultations. A recent study has reported that acceptance of telemedicine use among patients with knee osteoarthritis (KOA) is low at only 37%.^7^ This indicates that among older patients, lack of familiarity with internet-based communications and a preference for face-to-face communication with their physician are major factors in their unwillingness to use telemedicine.^7^

Another study described that anxiety regarding information technology and resistance to changes in patients' lifestyles were significant negative factors in the adoption of telemedicine.^8^ Expanding the use of telemedicine requires reducing patients' resistance to its use as well as enhancing the acceptance of telemedicine use, especially among older patients.

To the best of our knowledge, no studies have investigated potential factors that can contribute to changes in the acceptance of telemedicine in an orthopedic practice. In this study, we hypothesized that a rapidly increasing number of patients with COVID-19 infection can trigger changes in attitudes regarding the acceptance of telemedicine use among older patients with KOA. To test this hypothesis, we conducted a follow-up study during the third pandemic wave of SARS-CoV-2 in Japan.

## Materials and Methods

### Study setting, participants, and design

We conducted a cohort study in which participants were sampled from among older patients with KOA who regularly consulted the same orthopedic surgeon at the specialized knee outpatient orthopedic department of our hospital and affiliated hospitals located in Tochigi Prefecture, Japan. Patients were treated by outpatient physiotherapy and prescription of several nonsteroidal anti-inflammatory drugs once a month. We assessed whether older patients with KOA change attitudes regarding the acceptance of telemedicine use between baseline and a follow-up survey. All patients had no experience using telemedicine until the study initiation, and they just expressed their willingness to use telemedicine in both surveys. Committee of our institute approved the study and waived the requirement for informed consent (IRB approval number: 20-131).

**FIG. 1.**
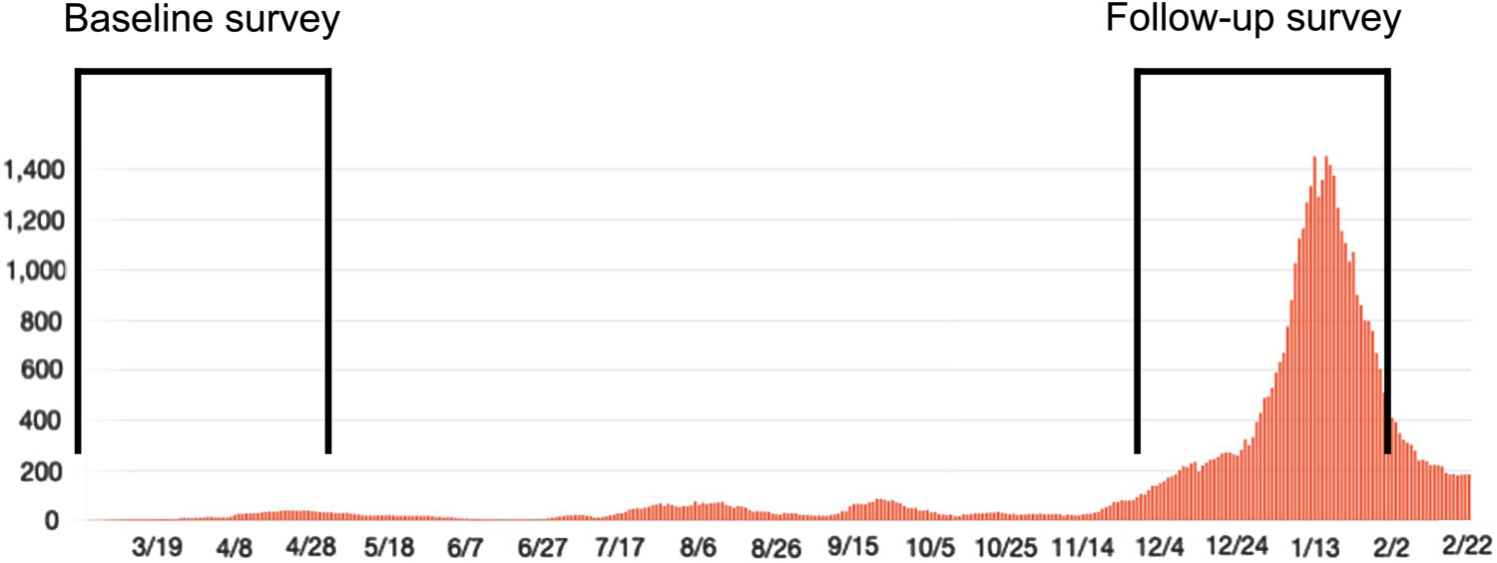
Baseline and follow-up survey periods and number of patients with coronavirus disease 2019 in Tochigi Prefecture, Japan. The date is the calendar year 2020.

We conducted a baseline survey from March 2020 to April 2020, and a follow-up survey was subsequently conducted from December 2020 to January 2021 (Fig. 1). An overview of the baseline survey has been previously published.^7^ During the follow-up survey period, many countries in the northern hemisphere, including Japan, experienced a third pandemic wave of SARS-CoV-2, which resulted in a spike in the number of patients with COVID-19 infection (Fig. 1).^9^

The third wave of the pandemic was particularly severe in the study area, Tochigi Prefecture, ranking among the top five most severely affected prefectures across Japan by population ratio between the baseline and follow-up surveys. We hypothesized that with the rapid surge in the number of patients with COVID-19 in a short time, the risk for hospital-related transmission of SARS-CoV-2 infection increased simultaneously, which could motivate patients to accept telemedicine use so as to avoid visits to the hospital.

### Measurements

We collected information regarding patients' demographic characteristics, including age, gender, smartphone use, time spent visiting a hospital (minutes), knee pain status, and severity of KOA. Knee pain status was measured using a numeric rating scale (NRS) for pain, which ranged from 0 (minimum) to 100 (maximum).^10^ The severity of KOA was based on findings regarding loss of range of motion and radiological findings per the Kellegren–Lawrence (KL) classification.^11^ In addition to demographic characteristics, we obtained information on patients' willingness to adopt telemedicine. All patients were asked whether they were willing to use telemedicine (Yes or No) in both baseline and follow-up surveys.

### Inclusion criteria

**FIG. 2.**
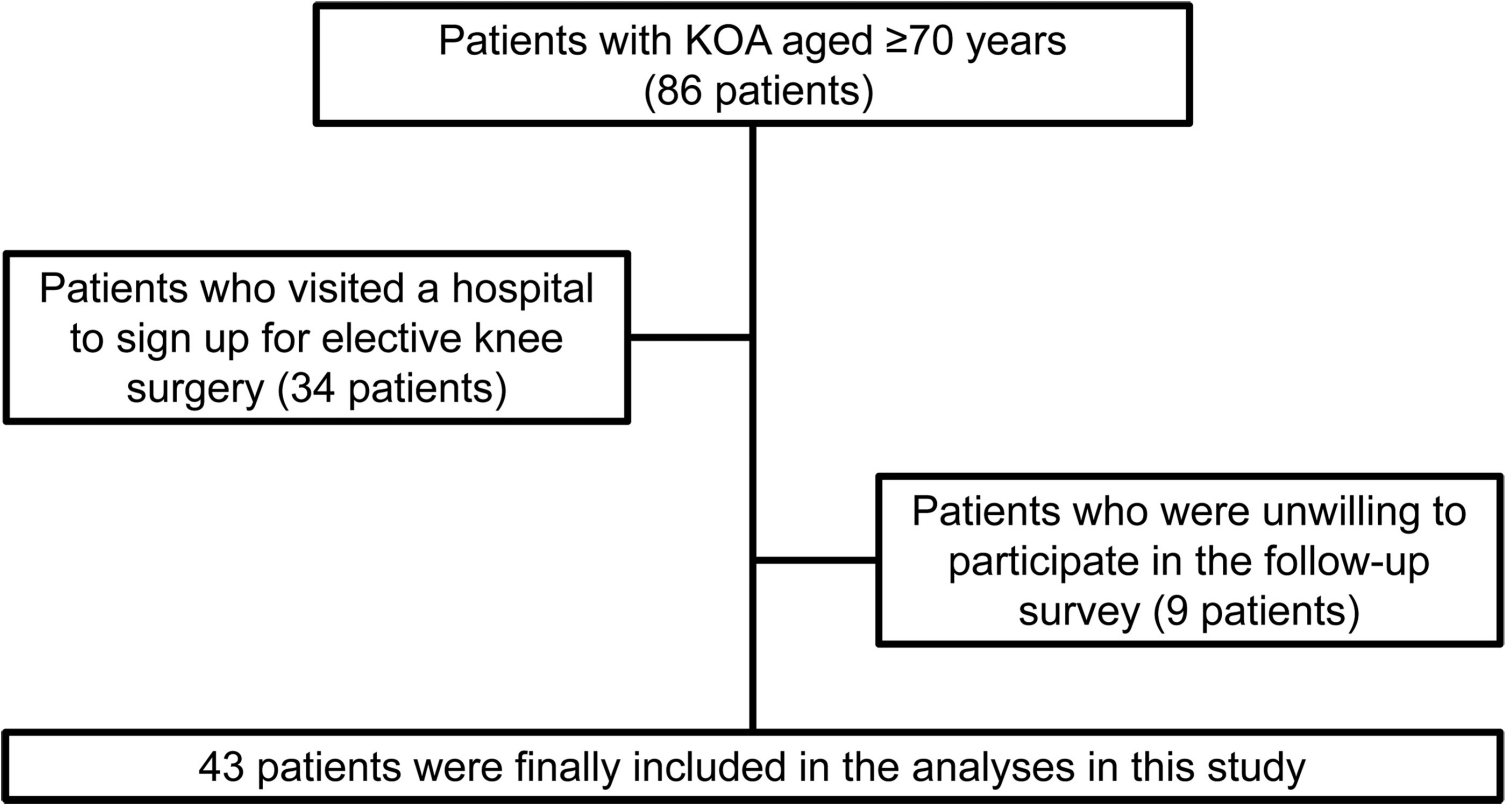
Patient selection. KOA, knee osteoarthritis.

The flow chart shows patient selection in this study (Fig. 2). The study only included patients with KOA aged ≥70 years.^7^ Patients who visited a hospital seeking elective knee surgery to alleviate pain were excluded from the analysis. Patients who were eligible in the baseline survey but were unwilling to participate in the follow-up survey were also excluded.

### Statistical analysis

First, we analyzed baseline survey data and described the distribution of demographic characteristics and the willingness to adopt telemedicine for all patients. Second, for willingness to use telemedicine in the baseline survey, we classified patients into two groups depending on whether they answered Yes (Group Y) or No (Group N). We then compared differences between these two groups. Finally, we assessed the change in willingness to use telemedicine as well as knee pain status between the baseline and follow-up surveys. In this analysis, the baseline NRS for knee pain was compared with the follow-up survey data. If the follow-up NRS was decreased from the baseline NRS, knee pain status was defined as improved. If the score was the same, knee pain status was defined as unchanged.

Numerical and categorical variables are presented as mean ± standard deviation and percentage, respectively. With the significance threshold at *p* < 0.05, numerical and categorical variables were compared using *t*-tests and chi-square tests, respectively. All statistical analyses were performed using EZR software.^12^ Jichi Medical University Bioethics Committee for Medical Research approved the study and waived the requirement for informed consent from individual participants (Approval ID: 20-131).

## Results

Patient characteristics at the baseline survey are given in Table 1. Of a total 43 patients with KOA, 12 (27.9%) were ≥80 years of age, with the maximum of 89 years; 15 (34.9%) patients were male; and 15 (34.9%) owned a smartphone. Patients spent a mean 24.0 min per hospital visit. The NRS ranged from 0 to 70, with mean score 25.3, and 18 (41.9%) patients were diagnosed as KL classification grade 4. Among the 43 patients, 11 (25.6%) stated that they would accept the use of telemedicine.

**Table 1.** Patient Characteristics at the Baseline Survey (*N* = 43)

Variables	*n* (%)
Age, mean ± SD, years	76.5 ± 5.2
70–79	31 (72.1)
80–89	12 (27.9)
Gender	
Male	15 (34.9)
Female	28 (65.1)
Smartphone possession	
Yes	15 (34.9)
No	28 (65.1)
Time required to visit hospitals, mean ± SD, min	24.0 ± 20.1
Numeric rating scale for pain, mean ± SD	25.3 ± 20.4
KL classification	
Grade 2	15 (34.9)
Grade 3	10 (23.3)
Grade 4	18 (41.9)
Willingness of adopting telemedicine	
Yes (Group Y)	11 (25.6)
No (Group N)	32 (74.4)

KL, Kellegren–Lawrence; SD, standard deviation.

Table 2 gives the comparison between Groups Y (*n* = 11) and N (*n* = 32) at the baseline survey. The percentage of smartphone ownership was significantly higher in Group Y than in Group N (63.6% vs. 25.0%, *p* = 0.020); however, other variables did not significantly differ between the groups.

**Table 2.** Comparison of Patients Based on Willingness to Use Telemedicine at the Baseline Survey (*N* = 43)

Variables	Willingness to use telemedicine		*p*
	Yes (Group Y), *n* = 11	No (Group N), *n* = 32	
Age (years old)	74.7 ± 5.2	77.1 ± 5.2	0.193
Gender (male/female)	3/8	12/20	0.539
Smartphone possession (yes/no)	7/4 (63.6%)	8/24 (25.0%)	0.020^a^
Time required to visit hospitals (min)	30.3 ± 28.7	21.8 ± 16.2	0.231
Numeric rating scale for pain	23.2 ± 16.2	26.0 ± 21.9	0.699
KL classification (Grades 2/3/4)	7/1/3	8/9/15	0.063

Data are presented as mean ± SD or number of patients. Numerical and categorical variables were compared using *t*-tests and chi-square tests, respectively.

^a^ Significant difference in the percentage of smartphone use.

All 43 patients completed the follow-up survey. No patients in Group N changed their response regarding willingness to use telemedicine. Thus, the follow-up survey resulted in the exact same number and percentage of patients who were willing to use telemedicine as in the baseline survey (Table 3). In contrast, NRS determined at the follow-up survey was lower than that at baseline, indicating that knee pain status improved during the study period (Table 3).

**Table 3.** Change in Willingness to Use Telemedicine and Knee Pain Status Between Baseline and Follow-Up Surveys (*N* = 43)

	Surveys		*p*
	Baseline (*N* = 43)	Follow-up (*N* = 43)	
Willingness to use telemedicine, *n* (%)			1.000
Yes	11 (25.6)	11 (25.6)	
No	32 (74.4)	32 (74.4)	
Numeric rating scale, mean ± SD	25.3 ± 20.4	22.6 ± 19.5	0.550

Percentages for willingness to use telemedicine were compared using chi-square tests, whereas the mean of the numeric rating scale was compared using *t*-tests.

Among patients in Group Y, 9.1% (1 of 11) reported improved knee pain at the time of the follow-up survey, whereas in Group N, 21.9% (7 of 32) reported improved knee pain (Table 4). A larger percentage of patients in Group N (21.9%) reported improvement in their knee pain than in Group Y (9.1%), although this difference was not statistically significant (*p* = 0.347). No patients in either group reported increased pain at the time of the follow-up survey.

**Table 4.** Change in Knee Pain Status Based on Willingness to Use Telemedicine (*N* = 43)

Knee pain status	Willingness to use telemedicine at baseline survey		*p*
	Yes (Group Y), *n* = 11	No (Group N), *n* = 32	
	*n* (%)	*n* (%)	
Unchanged	10 (90.9)	25 (78.1)	0.347
Improved	1 (9.1)	7/32 (21.9)	

Knee pain status was based on a numeric rating scale. Chi-square test was performed.

## Discussion

This was the first study to assess whether the rapidly increasing number of patients with COVID-19 affected changes in the acceptance of telemedicine in an orthopedic practice. In this study, we found that the proportion of patients who were willing to use telemedicine at the baseline and follow-up surveys did not differ. This result highlights that older patients with KOA did not change their willingness to accept the use of telemedicine, even with a drastically increased risk for hospital-related transmission of SARS-CoV-2 when visiting a hospital.

Notably, the present results also showed that 22% of patients in Group N (i.e., those who were unwilling to use telemedicine) reported improvement in their knee pain status at the follow-up survey. These findings indicate that acceptance of telemedicine among older patients may not be less sensitive to external environmental factors but rather telemedicine acceptance may be more sensitive to patients' personal factors, such as anxiety about information technology and resistance to changes in their lifestyle,^8^ regardless of improvement in patients' health condition.

We found persistently low acceptance of telemedicine in geriatric orthopedics, despite the drastically increasing number of patients with COVID-19 infection in Japan. Internet technologies provide a method for continuously recording people's biomedical signals, which may enhance the diagnosis and treatment of users' health conditions, such as in the field of cardiology.^13^ This development could contribute to decreasing the risk of hospital-related asymptomatic transmission of SARS-CoV-2 amid pandemic waves. However, the design of existing wearable technologies is not based on older adults' needs and considerations. Our previous study indicated that unfamiliarity with internet-based communication and a preference for face-to-face communication with medical doctors were the two main reasons that older patients with KOA did not accept telemedicine.^7^

Technology anxiety could affect older adults' willingness to use wearable technologies,^13^ and this tendency was in accordance with our previous and present results.^7^ Understanding older adults' individual lifestyles and their needs in all aspects can decrease anxiety toward future medical technologies, such as telemedicine in orthopedics. This study clarified that a rapid increase in the number of patients with COVID-19 infection did not affect older patients' willingness to use telemedicine, despite an increase of >35 times compared with the number of patients during the first wave of the COVID-19 pandemic, thus highlighting the strong anxiety of changing routine habits of outpatient visits among older people.

This study had several limitations. First, telemedicine use was not formally introduced yet in our study settings at the time of both baseline and follow-up surveys. Patients in our study had just expressed their willingness to use telemedicine. Second, the study was conducted in a limited number of hospitals located in the same prefecture and included a small sample size. Studies with a larger number of patients from various hospitals are warranted to further confirm our findings. Third, we only analyzed patients with KOA. Therefore, our findings may not be generalized to all patients with other musculoskeletal disorders.

## Conclusion

The acceptance of telemedicine use among older outpatients with KOA did not change, despite a drastically rapid increase in the number of patients with COVID-19 infection in Japan, which may lead to a drastically increased risk for hospital-related transmission of a potentially fatal infectious disease when visiting a hospital. The acceptance of telemedicine in older patients may not be less sensitive to the external environmental factors, but, instead, may be more sensitive to patients' personal factors, such as anxiety about information technology and resistance to changes in their lifestyle.

## Abbreviations Used

COVID-19: coronavirus disease 2019
KL: Kellegren–Lawrence
KOA: knee osteoarthritis
NRS: numeric rating scale
SARS-CoV-2: severe acute respiratory syndrome coronavirus 2
SD: standard deviation
